# Supplementary material for: Characterization of the anti-pathogenic, genomic and phenotypic properties of a Lacticaseibacillus rhamnosus VHProbi M14 isolate
Source: PLoS One. 2023 May 15;18(5):e0285480. doi: 10.1371/journal.pone.0285480 (PMC10184941; doi:10.1371/journal.pone.0285480)
Supplement: Attachment — Submitted filename: Response to Reviewer4.docx [file pone.0285480.s009.docx]

Reviewer #4:

Introduction

Line 44: “and produce lactic acid ….”

Line 47-51: The information on other genera is irrelevant to the main context as you did not investigate these bacteria and their interbacterial relationship in the current study.

Line 60- 61: Reference.

Line 67: “in the prevention of oral diseases…”

Line 69: “a strong inhibition effect” or “the strongest inhibition effect”

M&M

The research question is well defined, relevant, and meaningful.

Line 85: where were the S. mutans strains from? Obtained from commercial providers or isolated by the authors?

Line 97: The provider of the “Big Dye TM Terminator Cycle Sequencing Ready Reaction kit”

Line 104-108: It’s good to see you have tested a wide range of pathogens that are related to various diseases. However, it would be better to provide more background information in the section of Introduction on these target pathogens.

Line 111: again, where did you get the strain of F. nucleatum?

Line 164: the reference for Oxford cup agar diffusion assay

Results

Line 247-249: what were those isolated LAB? Why did not show the result?

Line 259-260: How close? What was the similarity?

Line 271-272: “because….” This sentence should be move to the discussion section.

Line 283: reductions of the adhesion index were significant

Discussion

Line 375: these bacteriocin-related genes were…

Line 376: Did you isolate these L. rhamnosus strains?

Answer:

1. Introduction. The manuscript had been revised.
2. M&M

Line 85: These pathogens are purchased from the BeNa Culture Collection Centre (Beijing, China) (Line 113).

Line 97: The manuscript had been revised.

Line 104-108: I will add the introduction of these pathogens in discussion.

Line 111: These pathogens are purchased from the BeNa Culture Collection Centre (Beijing, China) (Line 113).

Line 164: The manuscript had been revised.

3. Results

Line 247-249: The isolates were first observed by colony morphology and microscopy as rod-shaped bacteria. Since the main focus of this paper was on strain M14, information on other strains has been omitted.

Line 259-260: the 16S rDNA sequence of the strain was uploaded to the NCBI database and the strain was found to be close to genus *L. rhamnosus* by BLAST. Similar sequences refer to 16s rDNA.

Line 271-272: The manuscript had been revised.

Line 283: The manuscript had been revised.

4. Discussion

Line 375: The manuscript had been revised.

These strains are from other companies. By BLAST, they had similar bacteriocin related genes with strain M14.
