# Supplementary material for: Characterization of the anti-pathogenic, genomic and phenotypic properties of a Lacticaseibacillus rhamnosus VHProbi M14 isolate
Source: PLoS One. 2023 May 15;18(5):e0285480. doi: 10.1371/journal.pone.0285480 (PMC10184941; doi:10.1371/journal.pone.0285480)
Supplement: Attachment — Submitted filename: Response to reviewer1 and editors 0413.docx [file pone.0285480.s011.docx]

**Question:** "I still wonder because authors said “Bacteriostatic activity test against other pathogens”, but that is just to test antibacterial effects not for whether static or bactercidal activity. So, authors should change that. Authors need to add SD in the figure."

**Answer:**

Sorry, I didn't fully understand the reviewer last time.

This supernatant inhibition test has been done before for part pathogens. *E. coli*, *P. acnes* and *S. enteritids* didn’t test. I have redone the inhibition tests on these three pathogens and have added the latest test data to the manuscript. The zone of inhibition in the fermentation broth ranged from 1 cm to 2.47 cm, while the zone of inhibition in the supernatant （static or bactercidal activity）was relatively smaller. Because the bacteria in the fermentation broth are still growing and producing antibacterial substances (organic acids or bacteriocins) as it spreads.

The supernatant has no antibacterial activity against *C. albicans* and *P. acnes*, presumably because these two pathogens require higher concentrations of inhibitory substances.

I also repeated the heat sensitivity test for the inhibiting substance (excluding the interference of organic acids) and revised the manuscript.

I have added SD to the figures.

I have also revised other errors in the manuscript.

I have added a legend/caption for figures 1, 2, 3, 4, 5 and 6 in my main document.
